# Enhancing Muscle Activation Monitoring with Wearable Vibrating Sneakers: An EMG System-Based Approach for Continuous Health Assessment

**DOI:** 10.3390/bioengineering12101060

**Published:** 2025-09-30

**Authors:** Doo-Hoi Goo, Min-Su Heo, Woo-Young Chung, Hyeong Ho Hong, Eun-Yeong Jeong, Ji-Hyuk Kim, Jae-Chan An, Hae-Joo Kang

**Affiliations:** 1 Department of Sports Science, Sungshin Women’s University, 2 Bomun-ro 34da-gil, Seongbuk-gu, Seoul 02844, Republic of Korea; gdh76@sungshin.ac.kr (D.-H.G.);; 2 OOZOOTECH Inc., Lead Smart Square Knowledge Industry Center, 325 Sandan-ro, Danwon-gu, Ansan-si 15426, Gyeonggi-do, Republic of Korea; 3 College of General Education, Chung-Ang University, 84 Heukseok-ro, Dongjak-gu, Seoul 06974, Republic of Korea; 4 Dr. Hong Gym, 4th Floor, 306-28, Cheyuk-ro, Uijeongbu-si 11666, Gyeonggi-do, Republic of Korea; drhonggym@gmail.com; 5 Department of Social Welfare, Seoul Christian University, Seoul 03422, Republic of Korea; ey7078@hanmail.net; 6 Department of Sports Science, Gwangju University, Gwangju 61743, Republic of Korea; jhkim@gwangju.ac.kr (J.-H.K.);

**Keywords:** whole-body vibration, ectromyograph, lower limb muscles, muscle activity

## Abstract

**Background/Objectives**: Whole-body vibration (WBV) has been widely studied for its effects on neuromuscular activation, circulation, and balance. This study investigates the effect of wearing frequency of vibrating sneakers (18 Hz) on lower limb muscle activation during walking and squatting in middle-aged Koreans (40–60 years old). The objective is to assess whether WBV footwear enhances muscle engagement in both active and sedentary individuals. **Methods**: A 16-week randomized controlled trial was conducted with 64 participants divided into exercise (walking 30 min, three times a week) and non-exercise groups. Each group was further divided into wearing and non-wearing vibrating sneaker subgroups. Muscle activation of the quadriceps and gastrocnemius was measured using surface electromyography before and after the intervention. **Results**: Participants wearing vibrating sneakers showed significantly increased quadriceps and gastrocnemius activation during squatting and walking compared to non-wearers (*p* < 0.05). The exercise group wearing WBV sneakers exhibited greater improvements in muscle activation than the non-exercise group, indicating that WBV enhances the benefits of regular walking. However, no significant differences were observed in some lower leg muscles, suggesting that WBV effects may vary based on movement type and muscle group. **Conclusions**: Findings suggest that WBV sneakers (18 Hz) can enhance muscle activation during dynamic movements, potentially offering a low-impact training alternative for improving lower limb strength. These results provide valuable insights for exercise professionals, rehabilitation specialists, and wearable sensor developers, highlighting the potential of WBV footwear in neuromuscular conditioning and injury prevention.

## 1. Introduction

The global COVID-19 pandemic has substantially heightened public awareness of health and wellness, contributing to a marked rise in participation in physical activities such as walking and hiking. In South Korea, the number of individuals engaged in trekking reached 31.69 million in 2021, representing 77% of the adult population—an increase from 71% in 2018. Similarly, the proportion of individuals participating in regular walking activities increased by 9.3%, reaching 68.7% of the population [1]. These patterns underscore a growing emphasis on physical activity as a strategy for promoting overall well-being and preventing lifestyle-related diseases.

Walking, as a fundamental form of aerobic exercise, is critical for maintaining cardiovascular health and musculoskeletal function. Current guidelines recommend that adults perform at least 150 min of moderate-intensity aerobic activity per week to sustain optimal health [2]. Compared with other structured exercises, walking is highly accessible, requiring no specialized equipment and minimal training, thus serving as an effective intervention for individuals with limited time or mobility constraints [3]. However, the benefits derived from walking can be influenced by footwear design, surface conditions, and supplementary mechanical stimuli such as whole-body vibration (WBV).

The feet play a vital role in postural stability and locomotor mechanics. They contain a dense network of peripheral nerves that facilitate proprioception, balance, and circulation. Despite being anatomically the farthest from the heart, the feet assist venous return and are often referred to as the “secondary heart” [4]. Footwear is integral to maintaining efficient biomechanics during walking and running by enhancing shock absorption, supporting posture, and preventing excessive loading on the lower extremities. In contrast, poorly designed footwear, or shoes lacking adequate stability and cushioning, may induce abnormal gait patterns, hyperpronation, and elevated stress on the Achilles tendon, ligaments, and ankle joints [5,6,7].

Recent advancements in exercise technology have introduced vibrating sneakers as an innovative approach to enhance muscle activation and circulation during walking. WBV, traditionally delivered through stationary vibration platforms, has been extensively studied for its capacity to stimulate neuromuscular function, improve proprioception, and augment muscle performance [8,9]. Evidence suggests that WBV may be particularly beneficial for populations with mobility impairments, such as older adults and stroke survivors, by providing additional mechanical stimuli that enhance muscle strength, circulation, and postural control [10,11].

A recent network meta-analysis by Liu et al. [12] comprehensively compared the effects of WBVT with different frequencies on balance ability in older adults. Including 25 RCTs with 1267 subjects, the study found that low-frequency WBVT (LF-WBVT, <30 Hz) significantly improved outcomes in the Timed Up and Go Test (TUGT) compared with placebo and traditional rehabilitation groups. Although medium- and high-frequency WBVT also showed trends toward improvements, only HF-WBVT ranked highest in cumulative probability for enhancing balance, suggesting that frequency selection is crucial for optimizing outcomes. Importantly, the findings reinforce that LF-WBVT is both effective and safe for older adults, consistent with recommendations for populations at higher risk of musculoskeletal strain. Complementing this, Yu and Kwon [13] demonstrated that a structured WBV exercise program significantly enhanced physical performance and postural balance in older adults, with notable improvements in lower-limb muscle strength, cardiovascular endurance, and dynamic stability tests such as OLS and TUG. Their findings further support the utility of WBV as an elderly-friendly intervention that is both effective and low-impact. Similarly, Chung et al. [14] showed that different WBV frequency–amplitude combinations, even under the same acceleration, elicited distinct neuromuscular adaptations in young adults, with high-frequency/low-amplitude protocols improving isometric and isokinetic strength, while medium-frequency settings enhanced both concentric and eccentric contractions. These results highlight that frequency–amplitude parameters critically shape WBV outcomes, reinforcing the rationale for selecting low-frequency wearable devices (e.g., 18 Hz sneakers) in middle-aged and older populations, where safety and adaptability are key considerations.

However, the physiological effects of wearable WBV devices, such as vibrating sneakers, remain relatively underexplored compared to those of traditional stationary WBV platforms. Existing research indicates that WBV can induce muscle relaxation at specific frequencies, with the most pronounced effect observed at 16 Hz [15]. Nevertheless, only a limited number of studies have examined how continuous WBV exposure during walking influences muscle activation patterns. Furthermore, the integration of WBV with dynamic movements, such as squatting, may further enhance strength development, as prior evidence has demonstrated that WBV combined with resistance training yields greater strength improvements than resistance training alone [8,16]. In addition, WBV has been associated with increased bone mineral density [17] and reductions in body fat percentage [18,19], positioning it as a promising modality for enhancing overall musculoskeletal health.

Despite these reported benefits, WBV is not without risks. Prolonged or excessive exposure to high-intensity vibrations has been linked to adverse effects on biological tissues, including neuromuscular fatigue and microtrauma [10]. Nevertheless, when appropriately applied, WBV has been shown to serve as an effective intervention for fall prevention in older adults, as it improves muscle coordination and balance [9]. Importantly, neuromuscular responses to WBV vary according to frequency, amplitude, and individual training status, with evidence suggesting that optimal muscle activation occurs at specific vibration frequencies. For example, a study comparing vibration frequencies of 8 Hz, 26 Hz, and 40 Hz reported significant improvements in muscle strength only at 26 Hz [20]. Moreover, WBV has demonstrated effectiveness in clinical populations; for instance, in individuals with shoulder instability, higher-frequency and higher-amplitude WBV enhanced serratus anterior activation [21].

Muanjai et al. [22] investigated how WBV frequency affects muscle stiffness in the medial gastrocnemius (MG) and soleus (SOL) during plantar flexor isometric contractions. Using shear wave elastography, they showed that 24 Hz WBV significantly increased MG shear wave speed (muscle stiffness) at rest, while SOL exhibited a small reduction during contraction. Additionally, subjective ratings indicated increased stiffness after 12 Hz, and pennation angle decreased in MG during contraction after 24 Hz WBV. These results suggest that frequency-specific WBV induces differential neuromuscular responses within the triceps surae muscles and that shear wave elastography may be a useful tool for assessing acute changes in muscle mechanical properties. Importantly, the findings highlight that gastrocnemius and soleus respond differently to WBV depending on vibration frequency and contraction state, reinforcing the need to analyze specific lower-limb muscles when evaluating WBV interventions.

Traditionally, resistance training has served as the primary modality for enhancing muscle strength and power. However, this form of training requires substantial physical effort and is associated with a heightened risk of injury, particularly among older adults and individuals with pre-existing musculoskeletal conditions. In contrast, whole-body vibration (WBV) has emerged as a lower-impact alternative capable of eliciting comparable neuromuscular benefits while minimizing injury risk. Consequently, WBV has attracted growing attention as a potentially safer and more efficient training strategy, offering a promising complement or substitute for conventional resistance-based interventions [8,23,24,25,26].

Recent literature highlights that the physiological responses to whole-body vibration training (WBVT) are frequency dependent, typically classified as low-frequency WBVT (LF-WBVT, <30 Hz), medium-frequency WBVT (MF-WBVT, ≥30 Hz to <45 Hz), and high-frequency WBVT (HF-WBVT, >45 Hz) [27,28,29]. Studies have suggested that low-frequency WBV (<30 Hz) is often recommended for older adults and clinical populations due to its safer profile and reduced risk of musculoskeletal strain [30,31]. Accordingly, the wearable vibrating sneakers used in the present study were designed to operate at 18 Hz, a frequency within the low-frequency range, in order to balance effectiveness in stimulating muscle activation with safety and long-term feasibility for middle-aged populations.

While prior research has extensively investigated the effects of WBV administered through stationary platforms, relatively few studies have examined how wearable WBV technologies—such as vibrating sneakers—affect muscle activation during dynamic movements like walking and squatting. To address this gap, the present study evaluated the impact of vibrating sneakers operating at 18 Hz on lower-limb muscle activation in both exercising and non-exercising middle-aged adults. Specifically, the study assessed whether wearing vibrating sneakers during walking and squatting enhances neuromuscular engagement compared with conventional footwear.

Moreover, some manufacturers have claimed that WBV training at 25 Hz can generate muscle activation equivalent to walking 10,000 steps within just seven minutes. However, such claims remain largely unsupported by empirical evidence and are often employed as marketing strategies, contributing to consumer confusion. This underscores the need for rigorous scientific evaluation of wearable WBV devices. In this study, participants were divided into an exercise group (walking for 30 min, three times per week) and a non-exercise group, each further stratified into subgroups wearing or not wearing vibrating sneakers set at 18 Hz. Over a 16-week intervention, muscle activity was measured to evaluate changes in neuromuscular activation during both walking and squatting.

By systematically examining the effects of vibrating sneakers in these conditions, this study contributes empirical evidence to the expanding field of WBV applications. The findings provide new insights into the efficacy and limitations of wearable WBV devices in enhancing neuromuscular function, offering valuable implications for athletes, fitness professionals, rehabilitation specialists, and wearable sensor developers.

## 2. Materials and Methods

### 2.1. Participants

This study recruited 64 adult participants between the ages of 40 and 60, all residing in Seoul, South Korea. Eligibility criteria required that participants be free of musculoskeletal disorders or injuries that could interfere with their ability to complete the study protocol. To minimize confounding factors, individuals who had been diagnosed with musculoskeletal diseases or who had undergone musculoskeletal surgery within the previous six months were excluded from participation.

Participants were allocated into two primary categories: an exercise group, which engaged in structured walking for 30 min three times per week, and a non-exercise group, which did not follow a regular walking regimen. Each category was further subdivided based on footwear condition: participants either wore vibrating sneakers (18 Hz) or conventional sneakers without vibration. The distribution of participants across groups was as follows:

- Exercise group
  Wearing vibrating sneakers (18 Hz): 15 participants (14 women, 1 man).
  Not wearing vibrating sneakers: 15 participants (13 women, 2 men).
- Non-exercise group
  Wearing vibrating sneakers (18 Hz): 15 participants (14 women, 1 man).
  Not wearing vibrating sneakers: 19 participants (16 women, 3 men).

This group allocation allowed the study to isolate the effects of wearable whole-body vibration (WBV) on muscle activity during walking and squatting, while controlling for the influence of habitual physical activity.

### 2.2. Research Procedure

Prior to participation, all subjects—and their guardians when applicable—received a detailed explanation of the study’s objectives, procedures, and potential risks. Written informed consent was obtained from each participant before data collection commenced. Participants were then randomly assigned to one of the four groups using a computerized randomization tool to ensure unbiased allocation [32].

The exercise group was instructed to perform supervised walking sessions for 30 min, three times per week. Within this group, participants were further allocated to either the vibrating sneaker condition (18 Hz) or the non-vibrating sneaker condition. Likewise, the non-exercise group was subdivided into participants wearing vibrating sneakers and those wearing standard sneakers, creating a balanced framework for comparison.

**Figure 1:**
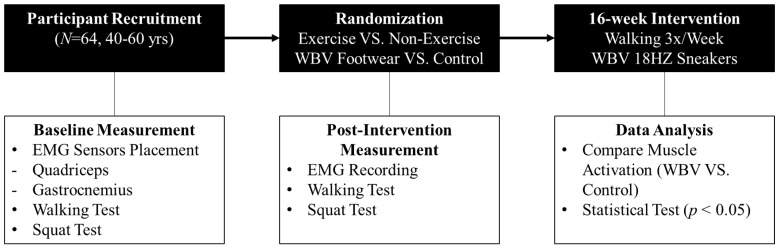
Research Procedure and EMG Measurement.

Neuromuscular adaptations were assessed at baseline and after 16 weeks of intervention. Muscle activation was measured during two primary tasks: walking and squatting. Surface electromyography (EMG) was employed to record activity in the quadriceps and gastrocnemius muscles both before and after the intervention period, allowing for a quantitative evaluation of changes in muscle activation under different conditions. Figure 1 illustrates the overall research procedure and EMG measurement.

The vibrating sneakers used in this study were custom-modified to incorporate a vibration circuit that delivers consistent stimulation at 18 Hz. A compact eccentric rotating mass (ERM) motor was embedded within the midsole region of each shoe to ensure direct transmission of vibration to the plantar surface. The motor was powered by a rechargeable lithium-ion battery integrated in the heel compartment, with wires concealed within the shoe body to maintain stability and comfort during walking. The vibration intensity and frequency were pre-programmed and fixed at 18 Hz to standardize stimulation across participants.

**Figure 2:**
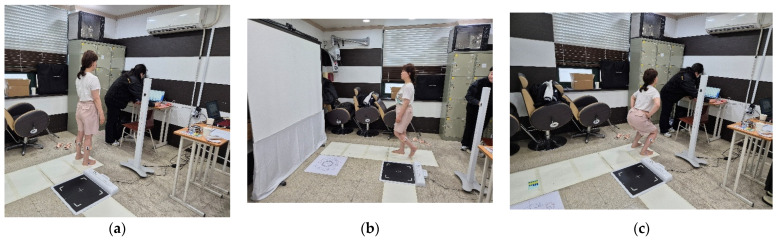
Experimental setup and measurement procedures. (**a**) Standing posture for baseline measurement, (**b**) walking trial across the gait analysis walkway, and (**c**) squatting task for dynamic muscle activation assessment. Surface EMG electrodes were attached to the quadriceps and gastrocnemius muscles to record neuromuscular activity in real time.

I confirm Figure 2 illustrates the experimental setup and procedures used to assess lower-limb muscle activation. Participants first stood barefoot on the gait analysis mat to establish baseline measurements, followed by a walking trial across the walkway to evaluate dynamic muscle activation during gait, and then performed squatting tasks to analyze neuromuscular responses during resistance-like movement. Surface EMG electrodes were strategically placed on the quadriceps and gastrocnemius muscles to capture electrical activity, while the system continuously transmitted data to the connected software for real-time monitoring and analysis.

### 2.3. Measurement

**Figure 3:**
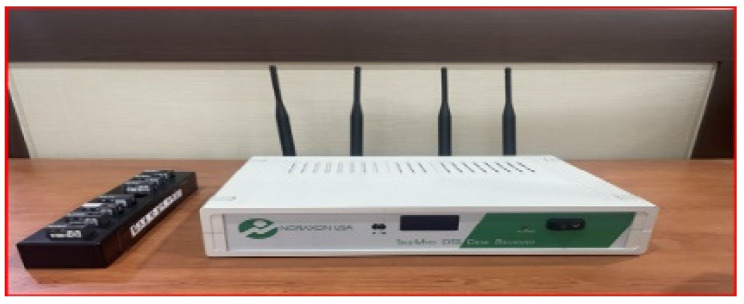
Electromyography device (Telemyo-DTS, NORAXON, USA).

Muscle activity was assessed using a wireless surface EMG system (TeleMyoDTS, Noraxon, USA) (Figure 3), which provides real-time recordings of electrical activity in the targeted muscle groups. This system was selected for its high reliability and non-invasive nature, making it suitable for capturing dynamic muscle activation during walking and squatting tasks.

To ensure methodological rigor and minimize measurement error, the following standardized procedures were implemented:

- Electrode placement: Bipolar surface electrodes were positioned at the midpoint of the quadriceps femoris and gastrocnemius muscles bilaterally (right and left legs).
- Skin preparation: Electrode sites were cleaned with alcohol swabs to reduce skin impedance, and hair was removed when necessary to ensure optimal signal transmission.
- Measurement conditions: All EMG measurements were conducted in a controlled laboratory setting with windows closed and curtains drawn to minimize environmental noise and external interference.
- Signal processing: EMG signals were collected at a sampling rate of 1000 Hz with a bandwidth of 40–450 Hz, and a 60 Hz notch filter was applied to eliminate electrical interference.
- Data processing: Recorded signals were processed using the root mean square (RMS) method to obtain a reliable measure of muscle activation.

Consistent with prior protocols in similar research, EMG data were analyzed as raw amplitudes (µV) without normalization to maximum voluntary contraction (MVC). This approach enabled a direct evaluation of changes in activation patterns across experimental conditions while avoiding potential variability introduced by MVC testing procedures.

**Figure 4:**
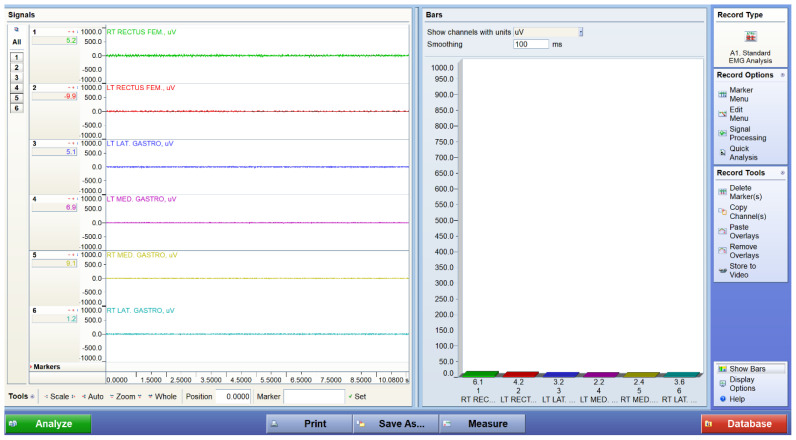
Representative EMG traces from a participant during baseline standing. Traces were acquired using a wireless surface EMG system (TeleMyoDTS, Noraxon, USA) from the rectus femoris and gastrocnemius muscles bilaterally.

**Figure 5:**
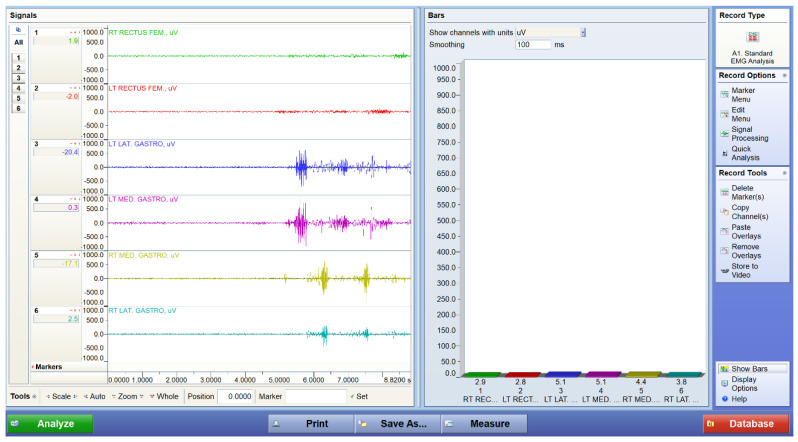
Representative EMG traces from a participant during walking. Traces were acquired using a wireless surface EMG system (TeleMyoDTS, Noraxon, USA) from the rectus femoris and gastrocnemius muscles bilaterally.

**Figure 6:**
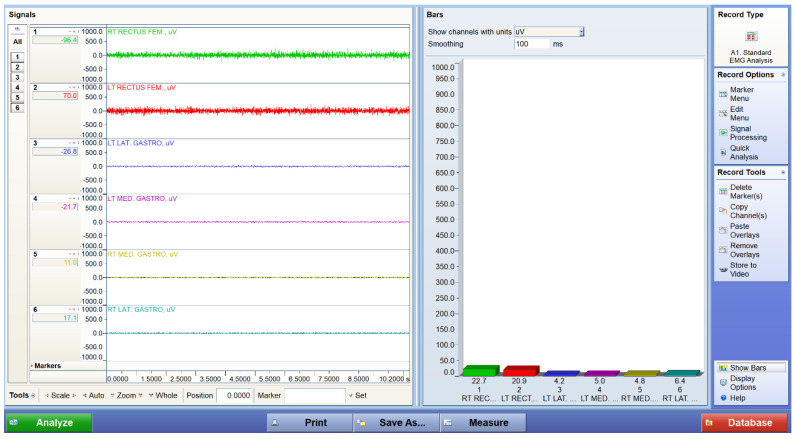
Representative EMG traces from a participant during squatting. Traces were acquired using a wireless surface EMG system (TeleMyoDTS, Noraxon, USA) from the rectus femoris and gastrocnemius muscles bilaterally.

Figure 4, Figure 5 and Figure 6 display representative EMG traces acquired from a participant under three experimental conditions: standing at baseline, walking, and performing squats. The baseline trace shows low-amplitude, stable electrical activity, confirming minimal background noise during standing. During walking, distinct bursts of activation are observed in both the quadriceps and gastrocnemius muscles, reflecting cyclical neuromuscular engagement in gait. Squatting produces sustained and higher-amplitude activation patterns, indicating greater muscle recruitment demands during resistance-like tasks. These EMG traces demonstrate the system’s ability to capture dynamic changes in muscle activation across different motor tasks.

### 2.4. Data Analysis

All statistical analyses were performed using SPSS (Version 29.0, IBM, USA), a widely used software for quantitative data analysis in science and biomechanics [33]. The dataset was first screened for missing values and outliers, ensuring data integrity and consistency before proceeding with statistical tests.

Measures of central tendency, including the mean (M) and standard deviation (SD), were calculated to summarize the distribution of key variables. To evaluate the effects of the intervention and determine significant changes in muscle activation before and after the 16-week period, paired *t*-tests were conducted within each group. The paired *t*-test is commonly used in repeated-measures designs to compare means from the same individuals under different conditions [34]. This test allowed for an assessment of within-group differences in quadriceps and gastrocnemius activation across the intervention period. Additionally, Pearson’s correlation analysis was employed to examine the relationships between muscle activation and other study variables, such as group assignment (exercise vs. non-exercise) and footwear condition (vibrating sneakers vs. non-vibrating sneakers). Pearson’s correlation is an essential tool for assessing the strength and direction of linear relationships between continuous variables [35]. The significance level for all statistical tests was set at α = 0.05, which is widely accepted as the threshold for meaningful interpretation in human movement research [33].

## 3. Results

### 3.1. Muscle Activity During Walking—Exercise Group

This section presents the analysis of muscle activity before and after walking in the exercise group, which was divided into participants wearing and not wearing vibrating sneakers (18 Hz) over a 16-week intervention (walking 30 min, three times per week).

As shown in Table 1, significant differences were observed in the quadriceps muscle after walking with vibrating sneakers. The right quadriceps muscle showed a decrease in muscle activity from 49.29 ± 38.06 to 24.48 ± 22.72 (*t* = 2.86, *p* = 0.012), while the left quadriceps muscle decreased from 40.08 ± 32.97 to 21.15 ± 20.30 (*t* = 2.61, *p* = 0.021). These results suggest that walking with vibrating sneakers may reduce the need for excessive muscle activation, potentially improving neuromuscular efficiency.

In contrast, no significant differences were found in the gastrocnemius muscle. The right gastrocnemius muscle changed minimally from 28.11 ± 8.67 to 28.24 ± 11.10 (*t* = −0.04, *p* = 0.969), while the left gastrocnemius muscle changed from 57.16 ± 28.27 to 58.03 ± 20.65 (*t* = −0.13, *p* = 0.891). Similarly, for the left leg, no statistically significant changes were found in gastrocnemius activation, indicating that vibrating sneakers may have a more pronounced effect on proximal rather than distal muscle groups.

For the group not wearing vibrating sneakers (Table 2), no significant differences were observed across all measured muscles. The right quadriceps muscle activity decreased slightly from 19.22 ± 12.26 to 17.74 ± 7.56 (*t* = 0.562, *p* = 0.583), while the left quadriceps muscle showed a non-significant decrease from 29.32 ± 21.47 to 21.52 ± 9.57 (*t* = 1.635, *p* = 0.124). The gastrocnemius muscles also exhibited minor fluctuations, but none reached statistical significance.

The results indicate that wearing vibrating sneakers (18 Hz) while walking significantly impacts quadriceps activation but does not affect gastrocnemius activation. This suggests that vibration exposure may alter neuromuscular recruitment patterns, particularly in the proximal muscles, potentially improving muscle efficiency. However, since no significant effects were observed in the gastrocnemius, future research should explore whether frequency adjustments or prolonged usage impact distal muscles differently. These findings provide preliminary evidence supporting the use of vibrating sneakers for lower-limb muscle activation modulation in walking-based exercise interventions.

### 3.2. Muscle Activity During Walking—Non-Exercise Group

This study examines the muscle activity before and after walking in the non-exercise group, which was divided into participants wearing and not wearing vibrating sneakers (18 Hz) over a 16-week period.

As shown in Table 3, participants in the non-exercise group who wore vibrating sneakers exhibited significant increases in muscle activation across multiple muscle groups. The right quadriceps increased from 27.79 ± 15.62 to 29.91 ± 13.23 (*t* = 2.153, *p* = 0.049), and the left quadriceps showed a similar increase from 22.66 ± 15.20 to 25.25 ± 13.62 (*t* = 2.270, *p* = 0.040). This suggests that even in the absence of an active exercise regimen, wearing vibrating sneakers can stimulate muscle activation during walking. In addition to the quadriceps, the gastrocnemius muscles showed notable activation changes. The right gastrocnemius muscle increased from 26.06 ± 10.11 to 30.11 ± 7.95 (*t* = 2.702, *p* = 0.017), and the left gastrocnemius muscle demonstrated a statistically significant increase from 25.32 ± 14.03 to 29.60 ± 12.77 (*t* = 2.626, *p* = 0.020). These results indicate that the application of whole-body vibration through footwear may enhance lower-limb muscle engagement even without structured physical activity.

For the group that did not wear vibrating sneakers (Table 4), the results were markedly different. Significant reductions in quadriceps activity were observed, with the right quadriceps decreasing from 42.76 ± 32.66 to 15.80 ± 8.17 (*t* = 3.809, *p* = 0.001) and the left quadriceps decreasing from 45.86 ± 34.81 to 14.75 ± 5.71 (*t* = 3.961, *p* = 0.001). These findings suggest that without vibration-induced stimulation, muscle activation during walking may decline over time in a non-exercise setting. Conversely, no significant changes were observed in the gastrocnemius muscles. The right gastrocnemius muscle showed a non-significant decrease from 30.08 ± 13.16 to 24.56 ± 9.01 (*t* = 1.392, *p* = 0.181), while the left gastrocnemius muscle remained relatively stable (*t* = 0.797, *p* = 0.436). This indicates that the absence of vibration exposure may primarily affect the quadriceps, whereas gastrocnemius activity appears less dependent on external stimulation.

The results suggest that wearing vibrating sneakers (18 Hz) can help maintain or increase lower-limb muscle activation even in individuals who are not actively exercising. This effect is particularly evident in the quadriceps and gastrocnemius muscles, supporting previous research indicating that whole-body vibration may enhance neuromuscular engagement. On the other hand, the decline in quadriceps activity observed in the non-vibrating sneaker group highlights the potential risk of muscle deconditioning in sedentary individuals. These findings may have implications for the development of passive exercise interventions aimed at preventing muscle decline in non-active populations.

### 3.3. Muscle Activity During Squat—Exercise Group

This section presents the results of muscle activity before and after the squat movement in the exercise group, which was divided into participants wearing and not wearing vibrating sneakers (18 Hz) over a 16-week period.

As shown in Table 5, participants who performed squats while wearing vibrating sneakers exhibited significant increases in quadriceps activation. The right quadriceps increased from 69.84 ± 27.27 to 79.90 ± 27.90 (*t* = 2.333, *p* = 0.035), while the left quadriceps showed a similar increase from 68.92 ± 31.69 to 89.20 ± 46.51 (*t* = 2.227, *p* = 0.043). These findings suggest that vibration exposure during squatting may enhance neuromuscular engagement, potentially improving lower-limb strength. Additionally, significant changes were observed in the gastrocnemius muscles. The left gastrocnemius increased from 7.63 ± 2.85 to 9.75 ± 4.01 (*t* = 2.681, *p* = 0.018), while the right gastrocnemius increased from 6.60 ± 2.69 to 7.50 ± 2.29 (*t* = 2.347, *p* = 0.034). The most substantial improvement was observed in the left gastrocnemius muscle (L), which increased from 8.36 ± 4.21 to 9.74 ± 3.77 (*t* = 3.428, *p* = 0.004), indicating a more pronounced response to vibration-induced stimulation.

For the group that did not wear vibrating sneakers (Table 6), the results were less pronounced. The right quadriceps showed a minor increase from 61.02 ± 21.07 to 63.56 ± 23.52 (*t* = 0.744, *p* = 0.469), while the left quadriceps increased from 63.68 ± 20.58 to 73.48 ± 30.39 (*t* = 1.763, *p* = 0.100). However, these changes were not statistically significant, suggesting that traditional squatting without vibration may not elicit substantial neuromuscular adaptations over the 16-week period. In the gastrocnemius muscles, only the left gastrocnemius (R) showed a significant increase from 7.45 ± 3.19 to 8.44 ± 3.51 (*t* = 2.270, *p* = 0.040). Other measurements, including right and left gastrocnemius (L), did not exhibit significant changes.

The findings suggest that wearing vibrating sneakers (18 Hz) during squat movements leads to significantly greater muscle activation, particularly in the quadriceps and gastrocnemius muscles. This aligns with previous research indicating that whole-body vibration can enhance neuromuscular stimulation, potentially improving strength and endurance. In contrast, the absence of vibration exposure resulted in limited improvements, particularly in quadriceps activation. These results highlight the potential benefits of integrating vibrating footwear into resistance training protocols to optimize muscle engagement during lower-limb exercises.

### 3.4. Muscle Activity During Squat—Non-Exercise Group

This section examines the muscle activity before and after squatting in the non-exercise group, which was divided into participants wearing and not wearing vibrating sneakers (18 Hz) over a 16-week period.

As shown in Table 7, significant changes were observed in quadriceps activation for participants who wore vibrating sneakers. The right quadriceps muscle exhibited a decrease in activation from 76.39 ± 37.49 to 68.54 ± 34.82 (*t* = 3.688, *p* = 0.002), while the left quadriceps increased from 78.89 ± 39.22 to 87.28 ± 36.39 (*t* = 2.497, *p* = 0.026). These findings suggest that vibration exposure may differentially affect muscle engagement, potentially enhancing neuromuscular coordination and efficiency. In contrast, the gastrocnemius muscles did not show statistically significant improvements. The right gastrocnemius increased slightly from 8.59 ± 3.68 to 9.69 ± 4.19 (*t* = 1.893, *p* = 0.079), while the left gastrocnemius increased from 6.65 ± 1.38 to 7.10 ± 2.40 (*t* = 0.867, *p* = 0.400). Similarly, no significant differences were found in the left gastrocnemius (L) (*t* = 0.546, *p* = 0.594) and right gastrocnemius (L) (*t* = 1.555, *p* = 0.142), indicating that whole-body vibration in a non-exercise setting may have a more limited effect on the lower-leg muscles.

For the group that did not wear vibrating sneakers (Table 8), no significant differences were observed across all measured muscles. The right quadriceps showed a slight increase from 68.14 ± 31.73 to 69.01 ± 29.76 (*t* = 0.226, *p* = 0.823), and the left quadriceps increased from 62.12 ± 21.62 to 68.09 ± 21.93 (*t* = 1.549, *p* = 0.139), but neither change reached statistical significance. Similarly, gastrocnemius activation remained relatively unchanged. The right gastrocnemius showed a non-significant change from 7.81 ± 5.49 to 7.51 ± 2.66 (*t* = 0.266, *p* = 0.793), while the left gastrocnemius exhibited a minimal increase from 7.35 ± 4.02 to 7.71 ± 3.24 (*t* = 0.579, *p* = 0.570). The left gastrocnemius (L) approached significance, increasing from 6.48 ± 2.76 to 7.70 ± 3.08 (*t* = 2.023, *p* = 0.058), but this result suggests only a marginal improvement.

The findings indicate that wearing vibrating sneakers (18 Hz) may enhance quadriceps activation during squat movements, even in individuals who do not engage in regular exercise. However, the lack of significant effects on the gastrocnemius muscles suggests that passive exposure to vibration alone may not be sufficient to induce lower-leg muscle adaptations. These results align with prior research emphasizing that whole-body vibration is most effective when combined with dynamic movements. The minimal changes observed in the non-vibrating sneaker group highlight the potential for muscle deconditioning in sedentary populations, reinforcing the importance of incorporating external stimulation methods, such as vibration-based interventions, to maintain muscle engagement.

## 4. Discussion

This study aimed to investigate the impact of wearing vibrating sneakers (18 Hz) on muscle activation during walking and squatting over a 16-week period. The participants were divided into four groups: an exercise group (walking for 30 min, three times per week) and a non-exercise group, with each group further divided into those wearing and not wearing vibrating sneakers. The study findings were analyzed in comparison with existing literature on whole-body vibration exercise, muscle activation, and neuromuscular adaptation.

WBV training has been widely studied for its effects on neuromuscular activation and performance enhancement. Previous research has suggested that WBV can stimulate muscle spindles and Golgi tendon organs, leading to reflex muscle contractions that improve strength, balance, and muscle endurance [36]. This mechanism is particularly useful for populations with limited mobility, as WBV can mimic traditional exercise effects without requiring significant voluntary movement [10]. Additionally, WBV has been shown to improve proprioception, which is crucial for balance and injury prevention [25]. The current study found significant differences in quadriceps activation in both the exercise and non-exercise groups when wearing vibrating sneakers, supporting prior findings that WBV can enhance lower-limb muscle activity [8]. This effect was particularly pronounced during squatting, where vibration stimulation likely increased neuromuscular recruitment and synchronization [37]. The significant activation of the quadriceps muscles aligns with previous findings that WBV is more effective for proximal muscles than distal muscles, likely due to differences in muscle fiber composition and neuromuscular control [17].

In the exercise group, wearing vibrating sneakers resulted in significantly reduced quadriceps muscle activation after walking. While this reduction may suggest improved neuromuscular efficiency—where less activation is required to achieve the same movement—we acknowledge that alternative explanations such as transient muscle fatigue or reduced motor unit recruitment cannot be completely ruled out. Our findings are consistent with prior studies reporting that WBV can reduce energy expenditure and optimize neuromuscular coordination during locomotor activities [38,39,40,41]. However, no significant changes were observed in gastrocnemius activation, suggesting that WBV may primarily influence proximal muscles during dynamic movements. The gastrocnemius muscle is heavily involved in plantarflexion and push-off during walking, but it may not experience the same level of vibration-induced activation as the quadriceps, which play a dominant role in knee extension and shock absorption [16]. These findings are consistent with prior research suggesting that WBV-induced adaptations are more pronounced in larger muscle groups with higher neuromuscular demand [11]. In contrast, in the non-exercise group, wearing vibrating sneakers led to significant increases in both quadriceps and gastrocnemius activation. This suggests that for individuals without regular exercise, WBV exposure may compensate for the lack of active training by enhancing neuromuscular activation [10]. The increased gastrocnemius activity in this group may be due to the absence of prior neuromuscular conditioning, which allows for greater adaptive responses compared to an active population [8].

During squatting, significant increases in quadriceps and gastrocnemius activation were observed in both the exercise and non-exercise groups wearing vibrating sneakers. The squat movement requires substantial lower-limb strength, particularly in the quadriceps and gastrocnemius muscles, which are responsible for knee extension and ankle plantarflexion, respectively [42]. The enhanced activation of these muscles aligns with studies indicating that WBV can increase muscle force production and improve postural control [37]. One possible explanation for these findings is the tonic vibration reflex (TVR), which occurs when vibration stimulates muscle spindles, resulting in enhanced motor unit recruitment and increased force output [36]. This mechanism has been shown to be particularly effective during resistance training exercises such as squats, where WBV can amplify muscle activation and improve overall performance [14]. Interestingly, while significant improvements were observed in the vibrating sneaker groups, the non-vibrating sneaker groups showed little to no changes in muscle activation. This finding supports prior research suggesting that traditional squatting without additional stimulation may not induce substantial neuromuscular adaptations over short- to medium-term periods [26]. In contrast, adding WBV appears to accelerate neuromuscular adaptations, likely due to increased afferent feedback and central nervous system involvement [9].

## 5. Conclusions

### 5.1. Theoretical Contribution

This study provides significant theoretical contributions by deepening our understanding of how WBV affects muscle activation during walking and squatting, particularly through the use of vibrating sneakers. Previous research has demonstrated the general benefits of WBV for muscle activation, strength, and balance improvement [8,9,43,44,45]. However, most studies have focused on traditional WBV platforms, overlooking how wearable WBV devices, such as vibrating sneakers, influence lower-limb muscle engagement in dynamic movements. While past studies have confirmed WBV’s effectiveness in enhancing neuromuscular activation during static exercises [37], they have not explored its effects during natural, everyday activities like walking. This study fills that gap by showing that the effects of vibrating sneakers vary according to exercise experience and movement type. In the exercise group, quadriceps activation decreased during walking, while gastrocnemius activation showed no significant change, suggesting that WBV may primarily influence proximal muscles in trained individuals. In contrast, in the non-exercise group, significant increases were observed in both quadriceps and gastrocnemius activation during walking. Furthermore, during squatting, vibrating sneakers enhanced activation of both muscle groups across exercise and non-exercise participants. These findings indicate that wearable WBV devices exert differential effects on lower limb muscle activation, highlighting the importance of user training background and activity context. Another critical distinction from previous work is the differential impact of WBV between active and non-active individuals. While earlier studies reported WBV-induced strength improvements [10,11], they did not distinguish between trained and untrained populations. This study revealed that non-exercisers experienced greater muscle activation changes than those already engaged in regular walking, suggesting that WBV may compensate for a lack of physical activity. These findings suggest new directions for WBV research. Scholars should further investigate how wearable WBV technology interacts with various movement patterns, load-bearing conditions, and long-term training adaptations. Additionally, research should explore whether frequency customization for different muscle groups could optimize neuromuscular efficiency. By addressing these gaps, future studies can refine WBV applications and expand its theoretical foundation.

### 5.2. Practical Implication

This study has valuable practical implications for fitness professionals, bodybuilders, sports trainers, sensor developers, and everyday users interested in enhancing muscle activation through wearable vibration technology. For fitness trainers and bodybuilders, the findings suggest that vibrating sneakers can be integrated into training programs to enhance quadriceps activation during squats. Since increased quadriceps activation was observed in both exercisers and non-exercisers, bodybuilders and strength trainers may benefit from incorporating vibrating sneakers into warm-ups, rehabilitation, or even resistance training routines to optimize neuromuscular efficiency. This is particularly useful for individuals recovering from knee injuries or those seeking alternative ways to stimulate muscle activation without increasing load. For sensor developers, this study highlights the importance of frequency optimization in WBV wearables. While prior WBV research has primarily focused on fixed-platform devices, this study suggests that wearable WBV can be customized to target specific muscle groups. Developers should explore adaptive vibration technology that adjusts frequency and amplitude based on movement patterns, user weight, and muscle engagement levels. For sports trainers, vibrating sneakers could be incorporated into injury prevention programs. Since WBV has been linked to improved proprioception and neuromuscular coordination [9], athletes recovering from lower-limb injuries may benefit from controlled exposure to WBV while performing functional movements such as walking and squatting. Lastly, general users, especially sedentary individuals or office workers, could use vibrating sneakers as a passive method to maintain muscle activation throughout the day. Given that non-exercisers in this study showed the most pronounced improvements, vibrating sneakers may be an effective tool for preventing muscle deconditioning in individuals with limited physical activity.

### 5.3. Limitations and Future Research Directions

Despite the promising findings, this study has some limitations. One key limitation is the lack of control for participant height and weight, which could influence plantar pressure and balance assessments. Studies have suggested that body mass plays a significant role in WBV-induced adaptations, as heavier individuals may experience different vibrational transmission effects compared to lighter individuals [17]. Future research should control for these variables using ANCOVA (analysis of covariance) to ensure that differences in muscle activation are not confounded by anthropometric factors. Furthermore, recent evidence shows that acute WBV effects may not always result in measurable changes in muscle stiffness or performance. For instance, Spadafora et al. [46] found that WBV did not significantly alter passive muscle stiffness or enhance countermovement jump performance in physically active males. This suggests that WBV outcomes may vary depending on participant characteristics and testing protocols, and highlights the importance of cautious interpretation when generalizing WBV effects. Additionally, the study did not examine long-term adaptations beyond the 16-week intervention. While WBV has been shown to produce acute neuromuscular benefits, its long-term effects on muscle hypertrophy, endurance, and injury prevention remain unclear [10]. Future studies should investigate whether continued WBV exposure leads to structural changes in muscle composition and function. Lastly, while this study focused on muscle activation, other physiological factors such as oxygen consumption, metabolic cost, and hormonal responses were not assessed. Prior research suggests that WBV may influence these variables, contributing to enhanced overall fitness and functional outcomes [42]. Incorporating these measures in future research could provide a more comprehensive understanding of WBV’s effects on human performance. Another limitation of this study is the absence of secondary performance data (e.g., heart rate, perceived exertion, or force output), which prevents ruling out alternative explanations such as fatigue. Future research should incorporate such measures to better validate whether reduced quadriceps activation reflects neuromuscular efficiency or other physiological mechanisms. Moreover, the gender imbalance in our sample, with a predominance of female participants, limits the generalizability of the findings to male populations. Future research should ensure a more balanced gender distribution to validate whether the observed effects of vibrating sneakers are consistent across sexes. Further, one limitation of this study is that muscle activity was analyzed using raw EMG amplitude values (µV) rather than normalized data (e.g., MVC), which makes direct cross-comparison with other studies more challenging. Future research should include normalization procedures and additional secondary performance measures to improve interpretability and validate whether observed changes truly reflect neuromuscular efficiency.

## Figures and Tables

**Table 1:** Muscle activity during walking before and after wearing vibrating sneakers (18 Hz) in exercise group (*n* = 15).

A		Pre-Test (µV)	Post-Test (µV)	*t*	*p*
		*M* ± *SD*	*M* ± *SD*		
quadriceps	R	49.29 ± 38.06	24.48 ± 22.72	2.86	0.012
	L	40.08 ± 32.97	21.15 ± 20.30	2.61	0.021
gastrocnemius (R)	R	28.11 ± 8.67	28.24 ± 11.10	0.04	0.969
	L	57.16 ± 28.27	58.03 ± 20.65	0.13	0.891
gastrocnemius (L)	R	51.20 ± 23.55	55.04 ± 18.04	0.61	0.546
	L	29.60 ± 10.48	28.85 ± 14.34	0.21	0.836

Note: Muscle activity values are expressed as raw surface EMG amplitude (µV).

**Table 2:** Muscle activity during walking before and after not wearing vibrating sneakers (18 Hz) in the exercise group (*n* = 15).

B		Pre-Test (µV)	Post-Test (µV)	*t*	*p*
		*M* ± *SD*	*M* ± *SD*		
quadriceps	R	19.22 ± 12.26	17.74 ± 7.56	0.562	0.583
	L	29.32 ± 21.47	21.52 ± 9.57	1.635	0.124
gastrocnemius (R)	R	19.09 ± 6.48	22.26 ± 9.43	1.537	0.147
	L	45.46 ± 16.90	42.37 ± 23.26	0.825	0.423
gastrocnemius (L)	R	34.34 ± 18.30	41.96 ± 16.90	1.785	0.096
	L	22.95 ± 11.26	24.47 ± 11.56	1.172	0.261

Note: Muscle activity values are expressed as raw surface EMG amplitude (µV).

**Table 3:** Muscle activity during walking before and after wearing vibrating sneakers (18 Hz) in the non-exercise group (*n* = 15).

A		Pre-Test (µV)	Post-Test (µV)	*t*	*p*
		*M* ± *SD*	*M* ± *SD*		
quadriceps	R	27.79 ± 15.62	29.91 ± 13.23	2.153	0.049
	L	22.66 ± 15.20	25.25 ± 13.62	2.270	0.040
gastrocnemius (R)	R	26.06 ± 10.11	30.11 ± 7.95	2.702	0.017
	L	38.66 ± 12.87	41.36 ± 14.42	1.614	0.129
gastrocnemius (L)	R	39.17 ± 16.22	45.87 ± 14.63	2.249	0.041
	L	25.32 ± 14.03	29.60 ± 12.77	2.626	0.020

Note: Muscle activity values are expressed as raw surface EMG amplitude (µV).

**Table 4:** Muscle activity during walking before and after not wearing vibrating sneakers (18 Hz) in the non-exercise group (*n* = 19).

B		Pre-Test (µV)	Post-Test (µV)	*t*	*p*
		*M* ± *SD*	*M* ± *SD*		
quadriceps	R	42.76 ± 32.66	15.80 ± 8.17	3.809	0.001
	L	45.86 ± 34.81	14.75 ± 5.71	3.961	0.001
gastrocnemius (R)	R	30.08 ± 13.16	24.56 ± 9.01	1.392	0.181
	L	50.39 ± 19.36	54.46 ± 26.99	1.048	0.309
gastrocnemius (L)	R	49.83 ± 20.08	52.03 ± 25.01	0.485	0.634
	L	29.84 ± 11.05	27.58 ± 9.36	0.797	0.436

Note: Muscle activity values are expressed as raw surface EMG amplitude (µV).

**Table 5:** Muscle activity during squat movement before and after wearing vibration sneakers (18 Hz) in exercise group (*n* = 15).

A		Pre-Test (µV)	Post-Test (µV)	*t*	*p*
		*M* ± *SD*	*M* ± *SD*		
quadriceps	R	69.84 ± 27.27	79.90 ± 27.90	2.333	0.035
	L	68.92 ± 31.69	89.20 ± 46.51	2.227	0.043
gastrocnemius (R)	R	9.83 ± 4.30	10.77 ± 3.49	1.880	0.081
	L	7.63 ± 2.85	9.75 ± 4.01	2.681	0.018
gastrocnemius (L)	R	6.60 ± 2.69	7.50 ± 2.29	2.347	0.034
	L	8.36 ± 4.21	9.74 ± 3.77	3.428	0.004

Note: Muscle activity values are expressed as raw surface EMG amplitude (µV).

**Table 6:** Muscle activity during squat movement before and after exercise group without wearing vibration shoes (18 Hz) (*n* = 15).

B		Pre-Test (µV)	Post-Test (µV)	*t*	*p*
		*M* ± *SD*	*M* ± *SD*		
quadriceps	R	61.02 ± 21.07	63.56 ± 23.52	0.744	0.469
	L	63.68 ± 20.58	73.48 ± 30.39	1.763	0.100
gastrocnemius (R)	R	10.02 ± 6.42	9.59 ± 5.56	0.443	0.664
	L	7.45 ± 3.19	8.44 ± 3.51	2.270	0.040
gastrocnemius (L)	R	8.15 ± 3.81	9.03 ± 4.44	1.158	0.266
	L	12.15 ± 7.33	10.13 ± 3.50	1.022	0.324

Note: Muscle activity values are expressed as raw surface EMG amplitude (µV).

**Table 7:** Muscle activity during squat movement before and after wearing vibrating sneakers (18 Hz) in the non-exercise group (*n* = 15).

A		Pre-Test (µV)	Post-Test (µV)	*t*	*p*
		*M* ± *SD*	*M* ± *SD*		
quadriceps	R	76.39 ± 37.49	68.54 ± 34.82	3.688	0.002
	L	78.89 ± 39.22	87.28 ± 36.39	2.497	0.026
gastrocnemius (R)	R	8.59 ± 3.68	9.69 ± 4.19	1.893	0.079
	L	6.65 ± 1.38	7.10 ± 2.40	0.867	0.400
gastrocnemius (L)	R	8.87 ± 5.68	9.12 ± 5.72	1.555	0.142
	L	9.78 ± 8.72	9.92 ± 8.60	0.546	0.594

Note: Muscle activity values are expressed as raw surface EMG amplitude (µV).

**Table 8:** Muscle activity during squat movement before and after not wearing vibrating sneakers (18 Hz) in the non-exercise group (*n* = 19).

B		Pre-Test (µV)	Post-Test (µV)	*t*	*p*
		*M* ± *SD*	*M* ± *SD*		
quadriceps	R	68.14 ± 31.73	69.01 ± 29.76	0.226	0.823
	L	62.12 ± 21.62	68.09 ± 21.93	1.549	0.139
gastrocnemius (R)	R	7.81 ± 5.49	7.51 ± 2.66	0.266	0.793
	L	7.35 ± 4.02	7.71 ± 3.24	0.579	0.570
gastrocnemius (L)	R	6.33 ± 2.66	7.19 ± 2.84	1.632	0.120
	L	6.48 ± 2.76	7.70 ± 3.08	2.023	0.058

Note: Muscle activity values are expressed as raw surface EMG amplitude (µV).

## Data Availability

The data supporting the findings of this study are available from the corresponding author upon request.
